# Epigenetic Regulation and Its Therapeutic Potential in Pulmonary Hypertension

**DOI:** 10.3389/fphar.2018.00241

**Published:** 2018-03-20

**Authors:** Yu Wang, Lingling Yan, Ziming Zhang, Eric Prado, Linchen Fu, Xuefeng Xu, Lizhong Du

**Affiliations:** ^1^Department of Pediatrics, Children's Hospital of Zhejiang University, Hangzhou, China; ^2^Loma Linda University School of Medicine, Loma Linda, CA, United States

**Keywords:** epigenetics, pulmonary hypertension, DNA methylation, histone modifications, noncoding RNAs, IUGR, EUGR

## Abstract

Recent advances in epigenetics have made a tremendous impact on our knowledge of biological phenomena and the environmental stressors on complex diseases. Understanding the mechanism of epigenetic reprogramming during the occurrence of pulmonary hypertension (PH) is important for advanced studies and clinical therapy. In this article, we review the discovery of novel epigenetic mechanisms associated with PH including DNA methylation, histone modification, and noncoding RNA interference. In addition, we highlight the role of epigenetic mechanisms in adult PAH resulting from undesirable perinatal environments—Extrauterine growth restriction (EUGR) and Intrauterine growth retardation (IUGR). Lastly, we give a comprehensive summary for the remaining challenges and discuss future methods of epigenetic targeted therapy for pulmonary hypertension.

## Introduction

**Figure 1:**
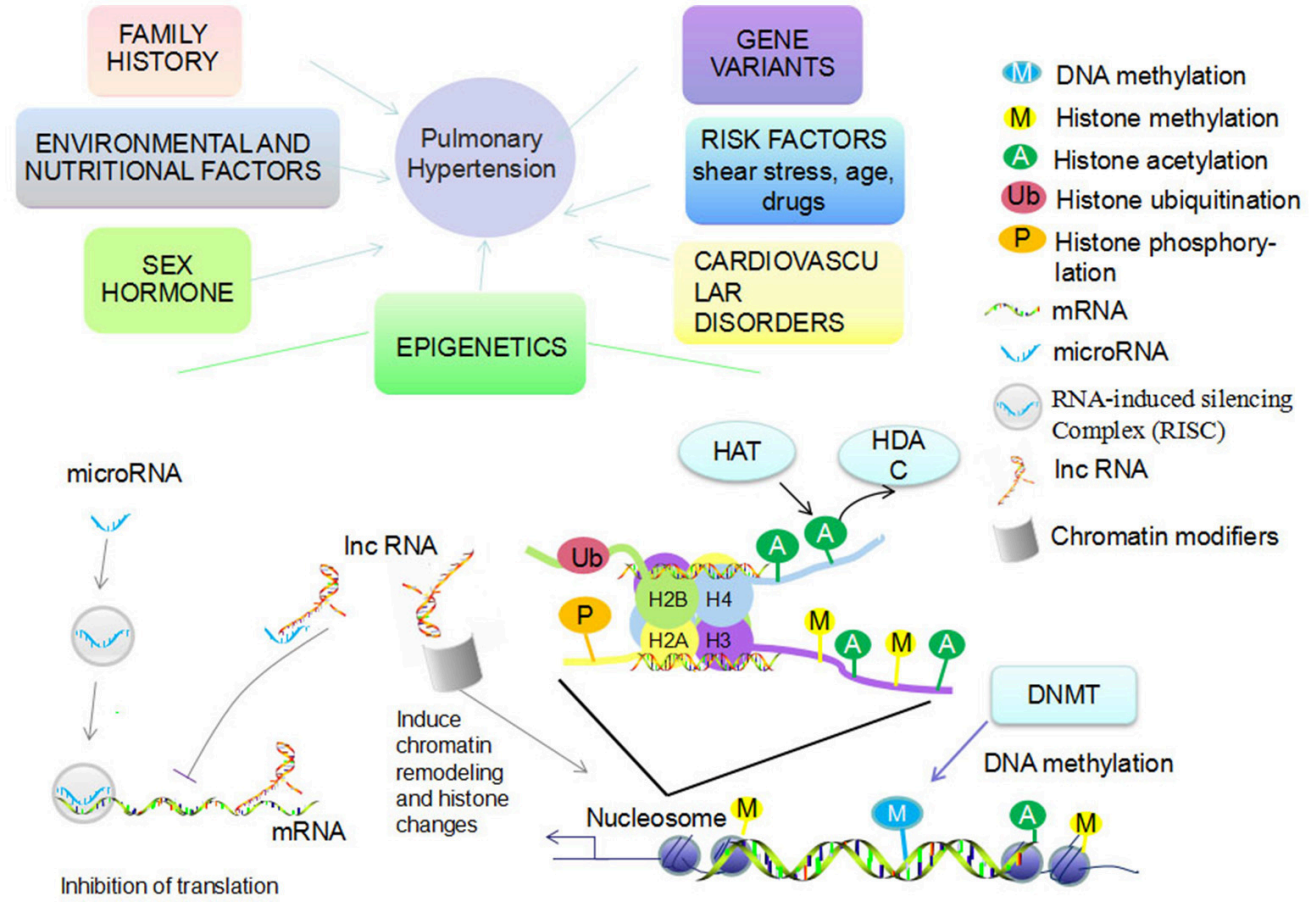
Etiologies account for PAH and epigenetic mechanisms. Multiple etiologies account for PAH, including gene variants, family history, levels of sex hormones, risk factors (shear stress, age, drugs), cardiovascular disorders, environmental (hypoxia, virus infection), and nutritional factors. Epigenetic mechanisms mainly include DNA methylation via DNA methyltransferase (DNMT) and histone modification (methylation, acetylation, ubiquitination, phosphorylation, etc.). Histone acetylation and deacetylation are regulated by histone acetyltransferase (HAT) and histone deacetylases (HDAC). The functional miRNA is incorporated into RNA-induced silencing complex (RISC) to silence gene expression, inhibit translation, or directly promote degradation of target mRNAs. LncRNA recruit chromatin modifiers while inducing chromatin remodeling and histone modifications. LncRNA can then either bind to mRNA to inhibit translation or bind to miRNA to inhibit RISC function.

Pulmonary arterial hypertension (PAH) is clinically defined as an elevation of mean pulmonary artery pressure (mPAP) >25 mmHg at rest (Barst et al., 2004). It is pathologically characterized by proliferation, migration, anti-apoptosis, or phenotype switching of pulmonary arterial endothelial cells (PAEC), pulmonary arterial smooth muscle cells (PASMC), and fibroblasts. The proliferation leads to an “onion-skin” appearance due to the occlusive lesions of small arteries which eventually become completely obstructed from the deposition of extracellular matrix (Edwards, 2004). Progressive remodeling of pulmonary vessels leads to a sustained increase in pulmonary vascular resistance (PVR) and afterload of right ventricle, which ultimately causes irreversible heart failure (Hyduk et al., 2005; Jongmin Kim, 2014). According to the 5th World Symposium of Pulmonary Hypertension held in 2013, PH can be classified into the five following groups: Group 1. Pulmonary arterial hypertension (PAH); Group 2. Pulmonary hypertension due to left heart disease; Group 3. Pulmonary hypertension due to lung diseases and/or hypoxia; Group 4. Chronic thromboembolic pulmonary hypertension (CTEPH); and Group 5. Pulmonary hypertension with unclear multifactorial mechanisms (Simonneau et al., 2013). Multiple etiologies account for PAH, including gene variants, family history, epigenetic changes, levels of sex hormones, risk factors (shear stress, age, drugs), cardiovascular disorders, environmental (hypoxia, virus infection), and nutritional factors (Figure 1; Kim et al., 2011; Tuder et al., 2013). Despite recent achievements in the treatment of PAH, most current therapies provide symptomatic relief for patients rather than cure the underlying disease. One of the challenges that confronts the successful reversing or curing of PAH is the consistent pulmonary hypertension that remains even after being treated maximally (Benza et al., 2012). Thus, there is an urgent need to explore the driving and sustaining molecular pathway of vascular remodeling as well as ventricular maladaptation in PAH for potential targeted therapeutic targets.

Epigenetics is the study of inherited changes in gene expression and phenotypic variance caused by mechanisms other than changes in DNA sequence (Saco et al., 2014). There are three main classes of epigenetic information that can be inherited over cell generations: DNA methylation, histone modifications, and non-coding RNA modification (Figure 1; Kim et al., 2011). As an interface between genetics and environmental influences, modification of epigenetic events play an important role in the onset and progression of different human diseases (Herceg, 2016). Thus far, studying epigenetics has enabled researchers to discover the mechanisms of cancer development and has led to FDA approved drugs that reverse epigenetic aberrations for cancer therapy (Manel, 2010). There is increasing evidence that there are important roles of epigenetic regulation in PH, however, the underlying molecular mechanisms remain to be elucidated (Kim et al., 2011). We explore the dysfunctional epigenetic mechanisms associated with PH in this review.

## DNA methylation and histone modification of PH

### DNA methylation of PH

DNA methylation refers to the covalent transfer of methyl groups (-CH3) onto the cytosine (C) pyrimidine ring in dinucleotide CpG (cytosine-guanine) DNA sequences (Bird, 2002). In mammals, it has been widely accepted that DNA methylation predominantly takes place on the fifth carbon of cytosine (5mC). Recently, another form of DNA modification, N6-methyladenine, has been identified in mammalian embryonic stem cells (Wu et al., 2016). DNA methylation is a physiological process that participates in the maintenance of gene activity states (genomic imprinting and silencing), cellular differentiation, and genome defense mechanisms (Paulsen and Fergusonsmith, 2001; Wu and Zhang, 2014). In human genomes, ~60–80% of CpGs within somatic cells are methylated, while the remaining are unmethylated. The unmethylated CpGs gather together to form a CpG-rich sequences known as CpG islands (Smith and Meissner, 2013). It is usually the unregulated hypermethylation of CpG islands that tends to be associated with aberrant transcriptions in cancer cells (Carvalho et al., 2012). This type of modification is set and maintained by several kinds of DNA methyltransferase (DNMTs) such as DNMT1, DNMT 3a, and DNMT 3b. DNA hypermethylation of promoters can frequently result in gene silencing by three of the following mechanisms: directly blocking the transcription factors from binding to specific DNA sequences, forming methyl-CpG binding proteins (MBP) to indirectly disrupt the recruitment of transcription factors, and silencing gene expression by cross-interference with histone modification (Ngo et al., 1995; Jones and Takai, 2001).

Superoxide dismutase-2 (SOD2) is a member of the SOD family, which controls the production of endogenous H_2_O_2_. H_2_O_2_ has the ability to regulate redox-sensitive targets, such as hypoxia inducible factor-1α (HIF1α) and voltage-gated potassium channel Kv1.5, both of which are important key factors that give rise to the mitochondrial role as a vascular O2 sensor (Sheehan et al., 1993; Eltzschig et al., 2005). SOD2 expression is decreased in PAH patients and fawn hooded rats (FHR). Archer et al. demonstrated that in pulmonary arteries of FHR, the selective hypermethylation of the SOD2 gene is located at both the enhancer region of intron 2 and a promoter region. Increased expression of DNMT1 and DNMT3B was also found. 5-aza-2′-deoxycytidine (Decitabine), a methyltransferase inhibitor, significantly decreased PASMC proliferation by demethylating SOD2, and increasing SOD2 expression (Archer et al., 2010). Recently, a genome-wide DNA methylation profile identified pulmonary endothelial cells genes of from PAH patients. A relationship between ABCA1 downregulation and lipid metabolism was shown which could be relevant to PAH (Hautefort et al., 2017). Reduced levels of global DNA methylation and histone acetylation were found in high-altitude long-term hypoxia induced PH. These epigenetic alterations may lead to PASMC proliferation and vessel remodeling (Yang et al., 2012a). Perros et al. demonstrated a decrease in gene Granulysin (GNLY) demethylation in peripheral blood mononuclear cells and explanted lungs in pulmonary veno-occlusive disease (PVOD) but not in PAH. This is helpful for discriminating these two diseases which share many similarities (Perros et al., 2013).

### Histone acetylation and histone methylation of PH

Histone modification is another key component of cellular regulation and it should always be considered in the topic of epigenetics. Histone modification is implicated in DNA repair processes, chromatin assembly, and genetic imprinting (Polo and Almouzni, 2005; Vidanes et al., 2005; Zhou et al., 2010). The fundamental building block of eukaryotic chromatins is the nucleosome which is comprised of positively charged histones that form an octamer (comprised of H2A, H2B, H3, and H4) with negatively charged DNA wrapped around them (Li, 2002). The N-terminal “tails” of the histones, especially the highly conserved sequences of H3 and H4, can undergo several kinds of post-translational covalent modifications including acetylation, methylation, ubiquitination, phosphorylation, biotinylation, glycosylation (*O*-GlcNAcylation), ADP-ribosylation, and SUMOylation (Bhaumik et al., 2007; Andreoli and Del, 2014). Histone acetylation enhances DNA transcriptional levels with histone acetyltransferase (HAT) which acts by transferring an acetyl onto the lysine (k) residues of histone proteins. The negatively charged acetyl group neutralizes the positively charged lysine residues, which loosens the binding between DNA and histone proteins. In contrast, histone deacetylation suppresses DNA transcription with histone deacetylases (HDAC) by removing acetyl groups from histones and subsequently tightening the chromatin (Eberharter and Becker, 2002; Verdone et al., 2005). The two processes mentioned above are active in a dynamic relationship depending on the balance between HAT and HDACs (Zentner and Henikoff, 2013). Currently, eight kinds of HAT are classified into two types according to their locations: nuclear or cytoplasmic. Eighteen kinds of HDAC have been discovered which can be classified into four groups (class I, IIa, IIb, III, and IV; Xu et al., 2007). Classification of histone deacetylases is listed in Table 1. Of all these HDACs, HDAC III is nicotinamide adenine dinucleotide (NAD+)-dependent also named as SIRT while the other three classes all have a zinc ion in the catalytic domains, which is required for catalysis (Ropero and Esteller, 2007). Previously, the small-molecule HDAC inhibitors are applied in the preclinical models of PH by chelating zinc in the active sites of HDAC I, II, and IV to increase the histone acetylation and promote the expression of specific genes (Cavasin et al., 2015). An emerging association between aberrant histone acetylation and PAH has been verified though various experiments. For example, there is an increased level of histone H3 and H4 acetylation at the promoter site of the endothelial nitric oxide synthetase (eNOS) gene in pulmonary arterial endothelial cells (PAEC) of persistent pulmonary hypertension of the newborn (PPHN; Xu et al., 2010). SIRT3, a mitochondrial deacetylase, is down regulated in human IPAH PASMC and rats with PAH. Decreased sirt3 suppresses mitochondrial function. It has also been found that sirt3 knockout mice can develop spontaneous PAH (Paulin et al., 2014).

**Table 1:** Classification of histone deacetylase (HDAC).

**Class**	**Name**	**Localization**
HDAC Class I	HDAC 1,2,3,8	Mainly nucleus
HDAC Class IIa	HDAC 4,5,7,9	Nucleus/Cytoplasm
HDAC Class IIb	HDAC 6,10	Mainly cytoplasm
HDAC Class III (SIRT)	SIRT 1,2,3,4,5,6,7	Nucleus/Cytoplasm/Mitochondria
HDAC Class IV	HDAC11	Nucleus/Cytoplasm

Several studies have discovered successful methods to cure PAH by using HDAC inhibitors to decrease arterial pressure. Sodium butyrate (BU), which acts as a histone deacetylase inhibitor, can inhibit PDGF-induced PASMC proliferation and migration (Cantoni et al., 2013). However, nonselective pan-HDAC inhibition with compounds such as TSA will bring about unwanted side-effects in the treatment for PAH and right ventricular (RV) hypertrophy. In Bogaard et al. experiment, treatment using the pulmonary artery banding (PAB) model with TSA did not reverse RV hypertrophy, but instead reduced cardiac output and increased RV dilatation (Bogaard et al., 2011). For this reason, selective HDAC inhibition has been considered to attenuate pulmonary hypertension. HDAC1 and HDAC5 are detected in higher expression in PAH patients and PAH rat models compared with the control group (Zhao et al.). Zhao et al. found that valproic acid (VPA) (an inhibitor to class I HDAC) and suberoylanilide hydroxamic (SAHA) (an inhibitor to classes I, II, and IV HDAC) are able to increase the acetylation of H3, reverse pulmonary hypertension, and reduce right ventricular hypertrophy with no effects to heart rate and systolic pressure (Zhao et al., 2012). Pulmonary adventitial fibroblasts, isolated from chronically hypoxic induced hypertensive calves, showed an accumulation of inflammatory cells and a significantly elevated catalytic activity of HDACs. The epigenetic associations can be illustrated when using class I HDAC inhibitors, which can markedly decrease cytokine/chemokine mRNA expression levels of the inflammatory cells (Courboulin et al., 2011). The class I HDAC inhibitor Apicidin can decrease right ventricular hypertrophy and remodeling of pulmonary arteries in PAEC of neonatal hypoxia induced PAH mice (Yang et al., 2015). The highly selective HDAC inhibitor for HDAC1, 2, 3 (HDACs class I)—MGCD0130 and MS275 were used in the hypoxia-induced PAH rat model to reduce pulmonary arterial pressure. A variety of beneficial effects on the right ventricle were seen though they only modestly reduced right ventricular hypertrophy (Cavasin et al., 2012). In PAH PAECs, binding of class IIa HDACs to Myocyte enhancer factor 2 (MEF2) results in suppression of MEF2 target genes that govern cellular growth and differentiation (Cavasin et al., 2015). Kim et al found that MC1568—pharmacological inhibition of class IIa HDACs, enables the restoration of impaired MEF2 activity and its targets such as miR-424 and miR-503 in PAH PAECs (Kim et al., 2013, 2015). In the monocrotaline (MCT) and Sugen/Hypoxia induced PH models, HDAC6 inhibitor Tubastatin A decreased PAH-PASMC proliferation and reduced mPAP (Boucherat et al., 2017).

The mechanism of HDAC inhibitors is complex. Generally, HDAC inhibitors increase histone acetylation and gene expression. However, HDAC inhibitors can also interact with non-histone substrates, such as transcription factors and coregulators, chaperones, nuclear receptors, nuclear import, signal transduction, DNA repair proteins, and mediators of movement (Marks and Xu, 2009). Therefore, based on these complex interactions, HDAC inhibitors can actually lead to reduced of gene expression. For example, NADPH oxidase 4 (Nox4) is involved in the development of PAH by contributing to reactive oxygen species (ROS) production, altered fibroblast behavior and cellular proliferation (Barman and Fulton, 2017). Nox4 expression is increased in the human PAH, hypoxia-induced, and MCT-induced PH model (Mittal et al., 2007; Barman et al., 2014). In the MCT-induced PH model, HDAC inhibitor VPA reduces right ventricular hypertrophy and rescues pulmonary hypertension by attenuating Nox expression (Chen et al., 2016). Current use of various epigenetic drugs in PAH are summarized in Table 2.

**Table 2:** Current use of various epigenetic modifiers in PAH.

**Mechanism**	**Drug**	**Targets**	**Function**	**Model**	**Cell type**	**References**
DNMT inhibitor	5-aza-2′-deoxycytidine	DNA methyltransferases 1 and 3B	mPAP↓	FHR rats with PAH	PASMC	Archer et al., 2010
Histone methyltransferase inhibitor	BX-01294	Histone lysine methyltransferase G9a for H3K9me2	P, M↓	PDGF induced proliferation	PASMC	Yang et al., 2012b
Histone demethylase inhibitor	GSK-J4	Histone demethylase JMJD3 for H3K27me3	P↓ Apoptosis↑	IPAH	PAEC	Gambaryan et al., 2013
HDAC inhibitors	Sodium butyrate	HDAC Class I (1,2,3,8)	P, M↓	PDGF induced PH	PASMC	Cantoni et al., 2013
	TSA	HDAC Class I/IIa/IIb/III/IV	RV dysfunction	PAB rats	–	Bogaard et al., 2011
	VPA	HDAC Class I (1,2,3,8)	mPAP↓, RVH↓	Human IPAH, hypoxia rats MCT-induced PAH	PASMC, PH-fibs, “R”-cells Isolated pulmonary arteries	Zhao et al., 2012 Chen et al., 2016
	SAHA	HDAC Class I/II/IV	mPAP↓, RVH↓	Human IPAH, hypoxia rats	PASMC, PH-fibs, “R”-cells	Zhao et al., 2012
	Apicidin	HDAC Class I (1,2,3,8)	RVH↓	Hypoxia mice	PAEC	Yang et al., 2015
	MGCD0130	HDAC Class I (1,2,3)	mPAP↓	Hypoxia rat	PASMC	Cavasin et al., 2012
	MS275	HDAC Class I (1,2,3)	mPAP↓, RVH↓	Hypoxia rat	PASMC	Cavasin et al., 2012
	MC1568	HDAC Class IIa (4,5)	RVSP↓	MCT, SUGEN induced PH, human PAEC	PAEC	Kim et al., 2013, 2015
	Tubastatin A	HDAC6	mPAP↓, RVSP↓	MCT, SUGEN induced PH, human PAH PASMC	PASMC	Boucherat et al., 2017

*DNMT, DNA methyltransferase; P, proliferation; M, migration; mPAP, mean pulmonary arterial pressure; PAB, pulmonary artery banding; RVH, right ventricular hypertrophy; RVSP, RV systolic pressure; “R”-cells:morphologically distinct cells potentially of hematopoetic origin with high growth potential (rhomboidal or “R”-cells); MCT, monocrotaline*.

Similar to DNA methylation, adding one (me1), two (me2), or three (me3) methyl groups into various basic residues of histones, usually targeted on histone H3, is the basic mechanism of histone methylation. On the same nucleosome, there is cross talk for linking DNA and histone methylation (Cheng and Blumenthal, 2010). For instance, methylation of H3K4 has been suggested to protect promoters from *de novo* DNA methylation in somatic cells to induce gene transcription (Weber et al., 2007). Conversely, methylation at H3K9 is positively correlated with DNA methylation and regarded as a code for transcriptional repression (Nguyen et al., 2002). The gene expression status is mainly determined by the site of a methyl lysine residue on the histone tail and the degree of methylation (me1, me2, or me3). Transcriptional modulator megakaryocytic leukemia 1 (MKL1) could interact with and recruit H3K4 methyltransferase complex in the hypoxia-induced pulmonary hypertension. Endothelial-specific depletion of two key components of the H3K4 methyltransferase complex reduces hypoxia-induced PH (Chen D. et al., 2015). Gambaryan et al. examined the expression of JMJD3, which can specifically demethylate H3K27me3 under the condition of cultured PAEC of PAH. It appears that GSK-J4, a selective JMJD3 inhibitor, can significantly lead to decreased proliferation, increased apoptosis and reduced TNF alpha-induced IL-6 release in a concentration-dependent manner (Gambaryan et al., 2013). Another similar experiment focused on the epigenetic regulatory effects of BX-01294, which is a specific inhibitor for G9a, a key enzyme for H3K9me3. It revealed that BX-01294 can also reduce PDGF-induced proliferation and migration of PASMC of pulmonary hypertensive ovine (Yang et al., 2012b). In the PASMC hypertensive mouse, an increased expression of Enhancer of Zeste Homolog 2 (EZH2), a mammalian histone methyltransferase, was detected. In transfected models, E2H2 can enhance proliferation, migration, and anti-apoptosis of the human PASMCs, compared to the controlled GFP-transfected cells (Aljubran et al., 2012).

## Epigenetic regulatory mechanisms of developmental origin of PAH

“Fetal origins of adult diseases” has gained increased attention in the past few years (Barker et al., 1989; Barker, 2004; Osmond et al., 2011; Szostakwegierek and Szamotulska, 2011). The original model of “the fetal origins of adult diseases” is “the Barker hypothesis.” It suggests that famine exposure during gestation sharply affects children's birth weight and even the susceptibility to diseases in adolescence and adulthood, including type 2 diabetes, impaired glucose tolerance, hypertension, coronary heart disease, metabolic diseases, and so on (Feng et al., 2015). In 2003, the academic community constructed the theory of “the Developmental Origins of Health and Disease” (DOHaD). Based on the theory of DOHaD, the beginning stages of life, including pregnancy, neonatal period, and childhood, are the crucial periods that may increase an individual's sensitivity or risk of developing diseases in adulthood (Barker and Osmond, 1986; Kubota et al., 2015; Dickinson et al., 2016).

Intrauterine growth retardation (IUGR) occurs during unsuitable uterine conditions which result in an average neonatal birth weight in the 10th percentile or 2 standard deviations lower than corresponding gestational age of fetus (Wu et al., 2006). According to a large amount of epidemic and lab research, IUGR is strongly correlated with the formation of adult-onset diseases (Vickers et al., 2000). Fetal tissues initiate some changes in order to adapt themselves to the unsuitable uterine condition. IUGR can lead to epigenetic changes of some related genes, eNOS, and endothelin-1 (ET-1), which make individuals hypersensitive to hypoxia, leading toward pulmonary arterial hypertension (Xu et al., 2013).

Endothelial nitric oxide synthetase (eNOS) catalyses the formation of NO—an endothelium derived relaxing factor which plays a vital role during the regulation of pulmonary arterial pressure. Histone modifications to different sites of eNOS promoter regions can make a difference for its activation or suppression. For instance, H3K9ace and H3K4me3 promote while H3K27me3 and H3K9me3 suppress the transcription of eNOS (Yan et al., 2010). Research based on the human endothelial cells isolated from umbilical veins (hUVEC) from control and IUGR fetuses uncovered the epigenetic mechanism underlying the eNOS changes (Krause et al., 2013). In IUGR-hUVEC, there is a decreased expression of eNOS associated with a hypermethylation of CpG-352 in its promoter. In addition, there is hypomethylation of the hypoxia response element (HRE) that occurs in the eNOS promoter region of IUGR-hUVEC, which is similar to the observation of normal hUVEC cultivated under hypoxic conditions (Casanello et al., 2009). More interestingly, silencing DNMT1 with siRNA against DNMT1 can reverse the eNOS expression and restore the response to hypoxia in hUVEC (Krause et al., 2013).

**Figure 2:**
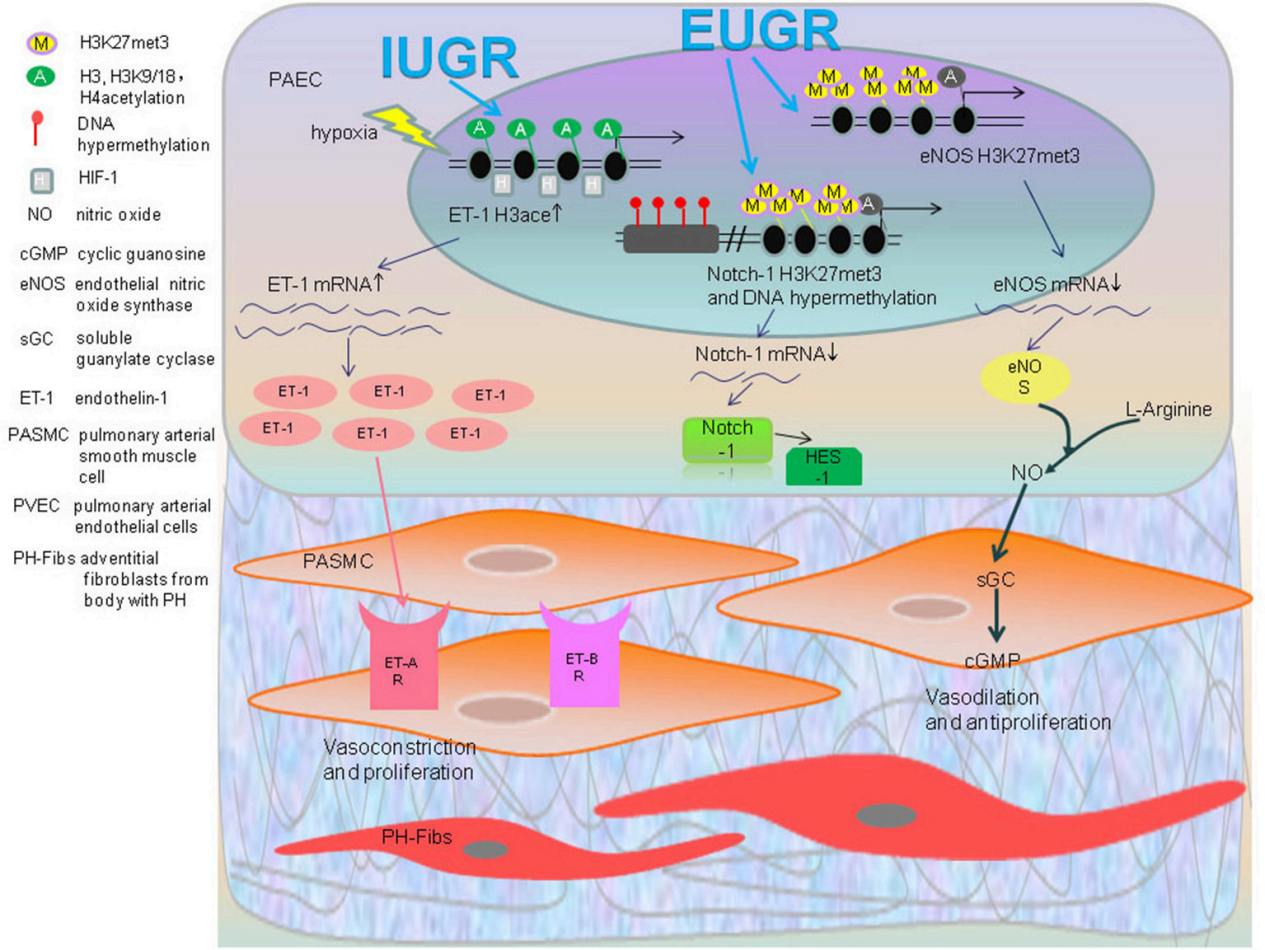
Epigenetic regulatory mechanisms of developmental origin of PAH: IUGR rats become hypersensitive to hypoxia as a result of PH along with an increased expression of ET-1, which is involved in contraction of pulmonary vascular. The acetylation of histone H3, H3K9/18, and H4 of ET-1 gene in IUGR-hypoxia rats PAEC accompanied with enriched transcription factor HIF-1α was found. The increased ET-1 activates ET receptors in PASMC that results in vasoconstriction and proliferation. In the EUGR induced PAH rat model, the levels of H3K27me3 of the eNOS gene were significantly higher. EUGR also caused H3K27me3 and methylation of a CpG site of Notch1 gene. This decreases the expression of eNOS mRNA, Notch1 mRNA and its downstream gene HES1 as a consequence, which impairs anti-proliferation and vasodilation.

Endothelin-1 (ET-1), a potent vasoconstrictor peptide, not only fosters the contraction of pulmonary vasculature, but can also stimulate the proliferation, migration, contraction and the deposition of extracellular matrix in vascular smooth muscle cells by activating ET receptors ((Kapakos et al., 2010)). When given a hypoxic stimulus, IUGR rats demonstrated a distinctly higher expression of ET-1 protein. Compared to the normal neonatal rats, the acetylation of histone H3 and H3K9/18 increases in core promoter regions of ET-1 gene in IUGR-hypoxia rats PAEC accompanied with the enriched transcription factor HIF-1α (Figure 2; Xu et al., 2013). Recently discovered, hyperacetylation is observed in the histone H3 in ET-1 promoters of leukocytes from the 1-week IUGR rats with a continued trend 10 weeks after birth (Xu et al., 2016). From this perspective, the epigenetic changes of peripheral leucocytes have a great potential to serve as a biomarker and risk predictor for developing disease (Xu et al., 2016).

Extrauterine growth restriction (EUGR) refers to the measured indicators of growth and development for neonates at the 10th percentile, which is currently inferior to expectations of intrauterine growth rate based on estimated postmenstrual age (Clark et al., 2003). One research study showed both systolic and diastolic blood pressures in adolescents born with EUGR history are dramatically higher than the control group (Ortizespejo and Mercedes, 2013). Another experiment demonstrated that providing IUGR rats with consistent EUGR feeding after birth will have a significantly higher pressure after 5 months (Tare et al., 2012). Hence, these results above indicate that EUGR is an inducer for aberrant functions of blood vessels for the future adult period. Higher pulmonary arterial pressure, increased right heart index, and remolding of pulmonary blood vessels can appear in adulthood of male EUGR rats, which is closely related to the epigenetic change of several key genes associated with PH (Zhang et al., 2014). Decreased expression of the eNOS gene in PAEC of EUGR male rats may arise from the increased presence of histone H3K27me3 and H3K9me3 in the proximal promoter region (Zhang et al., 2014).

Zhang identified that in PAEC of 9-week old EUGR male rats, there is an increased IGF-1 mRNA level, which may have resulted from decreased methylation in PAEC CpG islands as compared to the control groups. In the same experiment, an increased H3K27me3 and H3K9me3 of the PPARγ gene result in a lower expression of its mRNA. This provides the evidence that adult rats with a history of EUGR may undergo remodeling of pulmonary vascular structure and function, making them more susceptible to pulmonary hypertension (Zhang et al., 2014).

Notch1 gene plays an important role for the angiogenesis and repairing of injured vascular endothelial cells caused by ischemia (Limbourg et al., 2005). After forming a complex with NICD and RBP-j, Notch1 binds to the downstream transcription regulatory regions and activates the Notch signaling pathway, mediating the formation, maturation, and remodeling of pulmonary vascularture (Taichman et al., 2002; Gridley, 2007; Kume, 2009). Notch1 can synergistically pair with the VEGF gene to activate PI3K/Akt, which not only promotes the proliferation and migration of PAEC, but also increases the expression of NO to regulate the dilation of vessels by phosphorylation and activation of eNOS (Shiojima and Walsh, 2002; Takeshita et al., 2007). There is a significant reduction in Notch1 along with its downstream gene Hes-1 in the PAEC of EUGR rats of 3w and 9w. This may be related to the increase of histone H3K27me3 in the proximal promoter regions of Notch1 and the DNA hypermethylation of the CpG site in the distal promoter regions (Tang et al., 2015).

In addition to dietary restriction, other adverse environmental conditions, such as smoking, hypoxia, and drugs, are all associated with many diseases in adulthood. Insulin-like growth factor-1(IGF-1) is vital to the growth and metabolism of individuals, especially for the perinatal period. IGF-1 knockout mice suffer devastating embryonic and postnatal growth retardation (Woods et al., 1996). In the aspect of promoting vascular growth, IGF-1 can inhibit cellular apoptosis by phosphorylating apoptosis protein BAD through the IGF-1-IRS-1-PI3K-IGFR-1R/AKT pathway, which has been found to be activated in hypoxia-induced PAH (Madonna et al., 2015). Recent research has discovered that in the PAEC of neonatal hypoxia induced PAH mice, there is aberrant elevated cytosine methylation at the CpG site (position 87857993) in the promoter region of the IGF-1 gene. In addition, the HDAC inhibitor apicidin can diminish right ventricular hypertrophy and remodeling of pulmonary arteries by decreasing the activation of hypoxia-induced IGF-1/Pakt^S473^ signaling pathway (Yang et al., 2015). This study suggests a new relationship between the HDAC and traditional IGF-1 signaling pathway in the regulation of hypoxia-induced PH.

## Noncoding RNAs

Noncoding RNAs (ncRNAs) are the transcription products of genes that lack the ability to be translated into proteins. Noncoding RNA genes include tRNAs, rRNAs, snoRNAs, microRNAs, siRNAs, snRNAs, exRNAs, piRNAs, and scaRNAs and the long ncRNAs.

## MiRNAs mechanisms of PH

MiRNAs are small endogenous noncoding RNA that usually consist of <22 nucleotides. They are transcribed by RNA polymerase II from segments of genomic DNA appearing as primary miRNAs (pri-miRNAs) which then form 60–70 hairpin liked nucleotides known as the precursor miRNAs (pre-miRNAs) with the regulation of Drosha-DGCR8 complex. Then, Exportin-5 assists the GTP-Binding nuclear protein in transporting the pre-miRNA out of the nucleus to form mature miRNAs. The functional miRNA strand is then incorporated into a RNA-induced silencing complex (RISC) to silence gene expression by binding to the 3′-untranslated regions (UTR) of mRNAs, thus inhibiting their translation or directly promoting degradation of the target mRNAs (Lai, 2002; Johar et al., 2015). Comprehensive roles of miRNAs have been confirmed in various cancers, both oncogenes and tumor-suppressors (Volinia et al., 2010). Their potential function in the pathogenesis of cardiovascular diseases and targeted therapies have also become a significant research topic (Sanoudou et al., 2012).

Caruso was the first to report abnormal expressions of miRNA in PAH: downregulation of mir22, mir21, mir30c, and let-7f (Caruso et al., 2010), which created new therapeutic targets for the potential treatment of PAH, of which latter researchers could work with. Paracrine micro-RNA-regulated crosstalk in pulmonary arterial endothelial cells and pulmonary arterial smooth muscle cells has been discovered, for instance miR-17/20a and miR-130/301(Bertero et al., 2014, 2015). It is unclear whether these regulations in the microRNA of endothelial or smooth muscle-based cells have a similar impact on the outcome of PH. However, it is conceivable that these interferences may act on similar progenitors, as endothelial cells can transdifferentiate into smooth muscle cells when stimulated with platelet derived growth factor(PDGF)-BB (Yamashita et al., 2000) or transforming growth factor (TGF)-β (Frid et al., 2002). Furthermore, emerging evidence depicts the concept that miRNAs are affected by regulations from DNA methylation and histone modification, which form an internal regulatory network of epigenetics (Kim et al., 2013, 2015). Alterations of the expressions in different PH models and acting signaling pathways are listed in the Table 3.

**Table 3:** MicroRNA mechanisms in PH.

**Name**	**Expression**	**Pathway**	**Function**	**Cell type**	**Model**	**References**
Mir-9	Up	HIF-1α-mir-9-MYOCD	P↑,PC	PASMC	Hypoxia PASMC	Shan et al., 2014
Mir-130	Up	Mir-130-PPARγ-STAT3-Mir204 Mir-130-PPARr-apelin-mir424/503-FGF2	P↑	PASMC PAEC	Hypoxia+ SU5416 mice	Bertero et al., 2014
Mir-124	Down Up	Mir-124-NFATC1/PTBP1/CAMTA1 PTBP1/MCP1-notch1/FOXO3/p21CHIP	P↑ PC	PASMC Fibroblast	Hypoxia mice Hypoxia mice Hypoxia+SU5416 mice	Kang K. et al., 2013 Wang et al., 2014
Mir-138	Up	HIF-1α-mir138-MST1	A↓	PASMC	Hypoxia rats	Li et al., 2013
Mir-145	Up	Mir145-ACE/DAB2/FSCN1	VR	PASMC	Hypoxia mice PH patients Mir-145 knockout mice	Caruso et al., 2012
Mir-190	Up	Mir190-KCNQ5	VR	PASMC	Hypoxia rats	Li et al., 2014
Mir-193-3p	Up	Mir-193-3p-IGF1R/ALOX5,12,15	p↑	PASMC	MCT-induced PH rats PH patients	Sharma et al., 2014
Mir-204	Down	Mir-204-SHP2-Src-STAB-NFAT	P↑, A↓	PASMC	Hypoxia rats MCT-induced PH rats hPASMC	Courboulin et al., 2011
Mir-206	Down	Mir-206-Notch3	P↑, A↓,M↑, PC	PASMC	Hypoxia mice	Jalali et al., 2012
Mir-210	Up	HIF-1α-mir210-MKP1/E2F3	P↑, A↓	PASMC	Hypoxia mice	Gou et al., 2012
Mir-328	Down	Mir328-PIM1	P↑, A↓,VC	PASMC	PDGFBB-induce-d PASMC	Qian et al., 2016
Mir-451	Up	Promot hPASMC to migrate	M↑	PASMC	Hypoxia mice	Grant et al., 2013
Mir-27a	Up	Mir-27a-PPAR-ET-1	P↑	PAEC	Hypoxia mice	Kang B. Y. et al., 2013
Mir-424/503	Down	Mir-424/503-FGF2/FGFR	P↑, M↓	PAEC	MCT rats Hypoxia+ SU-5416 mice	Kim et al., 2013
Mir-17/92	Up —	IL6-STAT3-Mir-17/92-BMPR2/PDL1M5-TGFβ/smad2/3	PC, P↑	PAEC PASMC	Hypoxia mice MCT mice Mir17/92 knockout mice	Brock et al., 2009 Chen T. et al., 2015
Mir-21	Up Down	Mir21-PDCD4/SPRY2/PPARα Mir-21-BMP/Rho/Rho-kinase	P↑, M↑ VR	PASMC PAEC	Hypoxia MCT mice	Sarkar et al., 2010 Caruso et al., 2010
Mir125a	Down	miR-125a/Mfn1 axis	P↑	PASMC	Hypoxia rats	Ma et al., 2017
MiR-221-3p	Up	MiR-221-3p- AXIN2	P↑	PASMC	SU5416-hypoxia rats PH patients	Nie et al., 2017
miR-214	Up	miR-214-PTEN	P↑	PASMC	COPD PH patients	Liu et al., 2017
miR-135a	Up	miR-135a-BMPR2	PC	–	Th2 antigen (OVA) and urban particulate matter(PM) induced PH mice	Lee and Park, 2017

*P, proliferation; PC, phenotype change; A, apoptosis; VR, vascular remodeling; M, migration; PPARγ, peroxisome proliferator-activated receptor γ; STAT3, signal transducer and activator of transcription 3; FGF2, fibroblast growth factor2; NFATC1, nuclear factor of activated T cells 1; PTBP1, polypyrimidine tract-binding protein 1; CAMTA1, calmodulin-binding transcription activator 1; MCP1, monocyte chemotactic protein−1; MST1, serine/threonine kinase; ACE, angiotensin-converting enzyme; DAB2, disabled-2; FSCN1, fascin actin-bunding protein1; KCNQ5, postassium voltage-gated channel subfamily KQT member 5 protein; IGF1R, Insulin like growth factor 1 receptor; ALOX5, arachidonates 5-lipoxygenase; SHP2, Src homology-2 domain containing protein tyrosine phosphatase2; MCT, monocrotaline; Notch3, notch homolog 3 protein; MKP1, mitogen-activated APLN protein kinase phosphatase 1; E2F3, transcription factorE2F3; PIM-1:Ser/Thr-protein kinase-1; APLN, apelin; BMPR2, bone morphogenetic protein receptor type II; PDLIM5, PDZ and LIM domain5; PDCD4, programmed cell death protein4; SPRY2, Sprouty 2; PPARα, peroxisome proliferatior activated receptor-α*.

The MiR-17/92 cluster consists of six mature miRNAs (miR-17, miR-18a, miR-19a, miR-19b, miR-20a, miR-92a; Tanzer and Stadler, 2004), which are transcribed simultaneously but have their own respective target mRNAs with different regulatory functions. Down-regulation of bone morphogenetic protein receptor type II (BMPR2) protein, yet not the mRNA in several PAH models (Takahashi et al., 2006; Morty et al., 2007) revealed a posttranscriptional regulatory process mediated by miRNAs exists. Cytokine interleukin(IL)-6 increases expression of miR-17/92 through constant activation of STAT3(signal transducer and activator of transcription 3) binding sites in its promoter region(C13orf25), which inhibits BMPR2 and consequently proliferates the endothelial cells (Brock et al., 2009). MiR-17/92 leads to the expression of contraction proteins of PASMC by suppressing PDZ and LIM domain5(PDLIM5) by binding to its 3′UTR, this protein then induces TGF-β/Smad3 signaling (Chen T. et al., 2015). Pullamsetti et al. reported that miR-17-92 exhibits an over expression in both hypoxia-induced and MCT-induced rat models. An inhibitor of miR-17 can ameliorate disease states in PH (Pullamsetti et al., 2012).

In PH-hPAEC, miR-27a is expressed more with ET-1 by reducing the expression of PPARγ (Kang B. Y. et al., 2013). The micro-130/301 family (miR-130a, miR-130b, miR-301a, and miR-301b) was found to be a master regulator of several subordinate miRNAs. Bertero et al. discovered that in PAECs, miR-130/301-PPARγ contains the same upstream regulatory axis of the APLN-miR-424/503-FGF2 axis that exerts cellular proliferation by repressing apelin and its respective miRNAs (miR-424/503; Kim et al., 2013; Bertero et al., 2014). This miR-130/301-PPARγ is involved in regulating STAT3-miR-204- Scr kinase—NFAT which are substances for the PH-PASMC proliferation and anti-apoptosis (Courboulin et al., 2011). As expressed, the up-regulatory miR130/301 family is of significant importance in the microRNA network of PH, which enables it to become a potential biomarker and antagonistic target for PH therapy (Bertero et al., 2015).

In PASMC isolated from hypoxic rats and patients, miR-124 was down-regulated. The action of miR-124 not only caused a significant decrease in cell proliferation but also maintained a differentiated phenotype by inhibiting its target—nuclear factor of activated T cells (NFAT) signaling pathway (Kang K. et al., 2013; Wang et al., 2014). Hypoxia stimulates the HIF1α induced up regulation of miR-9, leading to the phenotypic switching of PASMC from contractile to proliferative (Shan et al., 2014). MicroRNA21 was found to be down regulated in idiopathic PAH patients' lung tissue and serum as well as MCT-induced PH rats (Caruso et al., 2010). In PASMC isolated from hypoxia induced PH mice, there is an increased expression of miRNA21 which promotes the proliferation and anti-apoptosis of PASMC by down regulating the following target genes: PDCD4, SPRY2, and PPARα (Sarkar et al., 2010). MiR-125a was found to decrease PASMC proliferation during hypoxia by inhibiting Mfn1 expression. MiR-125a agomir had a protective role in mitochondrial dysfunction and abnormal remodeling (Ma et al., 2017). MiR-138 was found to represses serine/threonine kinase Mst1 as a result of activating the Akt signaling pathway, thus functioning as a negative regulator for the apoptosis of PH-PASMC by a mitochondria-mediated caspase-dependent mechanism (Li et al., 2013). Caruso et al. found an elevation of miR-145 expression in PH-PASMC, especially in the lungs of BMPR2-deficient mice and patients with BMPR2 mutations (Caruso et al., 2012). With this current knowledge, the development of a patent for using antisense oligonucleotides (ASOs) against miR-145 has been initiated in chronic hypoxia mouse models, which provides a promising approach for treating the progression of PH (Ogorodnikova and Arenz, 2015). In PH-PASMC, increased miR-190 enhances vasoconstriction by interacting with its target Kcnq5, a subfamily member of voltage-gated K^+^ channels, which mediates Ca^2+^ influx (Li et al., 2014). Sharma et al. found that in PAH patients and rodents, miR-193-3p was significantly down regulated. This is due to 4F peptide, an Apolipoprotein A-I (apoA-I) mimetic peptide, which can restore the expression of miR193 by suppressing lipoxygenases (ALOX5,12,15), IGF1R and retinoid X receptor (RXR) α signaling pathway (Sharma et al., 2014). Over-expression of miR-206 in hPASMC can decrease proliferation, increase apoptosis and induce differentiation of α-SMA and calponin by inhibiting Notch3 protein expression (Jalali et al., 2012). MiR-221-3p promotes proliferation of PASMC by targeting AXIN2. This proliferation can be dampened by miR-221-3p inhibitors, AXIN2 over-expression, or β-catenin inhibition (Nie et al., 2017). MiR-210 is an HIF1α-dependent miRNA that can inhibit PASMC apoptosis during hypoxia by directly repressing E2F3 expression (Gou et al., 2012). MiR328 is down regulated in PASMC and functions as an inhibitor of PASMC's proliferation and migration by targeting the Ser/Thr-protein kinase-1 (PIM-1). Based on these findings, measuring serum miR-328 levels is a promising diagnostic tool for CHD-PAH diagnosis (Qian et al., 2016). Over-expression of miR-451 under serum-free conditions only promoted hPASMC to migrate. Temporary inhibition of miR-451 can reduce the development of PAH but genetic deletion of miR-451 had no beneficial effects on PAH (Grant et al., 2013). Increased miR-214 was found to promote hypoxia-induced PH with COPD by targeting PTEN in PASMCs. As such, miR-214 could be a potential circulating biomarker and novel therapeutic target in the treatment of PH (Liu et al., 2017).

Wang et al. demonstrated that in the PH fibroblasts, miR-124 is inhibited with its downstream genes such as Nothch1, PTEN, FOXO3, p21, and p27 by activating polypyrimidine tract-binding protein (PTBP)-1. In addition, there is also increased inflammatory activity associated with miR-204 in PH fibroblasts when searching for its direct target MCP-1—a chemoattractant for macrophages and monocytes, which was shown to have increased levels when transfecting anti-miR-124 (Wang et al., 2014). The study reveals multiple targets (PTBP1 inhibitors, miR-124 agonists) with high therapeutic potential for IPAH patients.

In a PAH model induced by Th2 antigen (OVA) and urban particulate matter (PM), miR-135a was significantly increased, and its target BMPR2 was decreased. MiR-135a could effectively serve as an additional biomarker. A drug that blocks the action of miR-135a could be useful for therapeutic intervention in PAH (Lee and Park, 2017).

## Long noncoding RNAs

Long noncoding RNAs (lncRNAs) are noncoding RNA molecules consisting of greater than >200 nucleotides. Recent evidence points to the involvement of lncRNAs in multiple biological processes including cell proliferation, cell growth, cell differentiation, cell cycle progression, apoptosis, immune response, imprinting, tumorigenesis, pathogenesis of various human diseases (Kogo et al., 2011; Wapinski and Chang, 2011; Ziats and Rennert, 2013; Vencken et al., 2014; Greco et al., 2015; Ling et al., 2015) and other biological processes (Ota et al., 2004; Tupy et al., 2005; Wilusz et al., 2009).

LncRNAs play important roles in epigenetic mechanisms in transcriptional and post-transcriptional regulation by targeting transcription factors, inducing chromatin remodeling, regulating methylation complexes, blocking proximate transcription (Ponting et al., 2009), and RNA based epigenetic regulatory networks through small interfering RNAs (si-RNA; Singh and Prasanth, 2013; Weinberg and Morris, 2013).

Despite the increased knowledge of lncRNAs, the epigenetic mechanisms of lncRNAs involved in PAH remain poorly understood. Recently, some studies have expanded our understanding of lncRNAs in PAH. Chronic thromboembolic pulmonary hypertension (CTEPH) is one of the primary causes of severe pulmonary hypertension. In endothelial tissues from the pulmonary arteries of CTEPH patients, aberrant expression of 185 lncRNAs was observed. The expression levels of lncRNAs, NR_036693, NR_027783, NR_033766, and NR_001284 were significantly altered. The potential role of lncRNAs may involve inflammatory responses, response to endogenous stimulus, antigen processing and presentation (Song et al., 2015). In the hypoxic pulmonary hypertension (HPH) rat model, the expression profile of 362 lncRNAs was significantly altered using a microarray (Wang et al., 2015). Extracellular circulating lncRNAs in human plasma were tested. However, the results showed no changes and very low levels of 84 lncRNAs in plasma of PAH. Therefore, the use of lncRNAs as a potential biomarker for human PAH is limited (Schlosser et al., 2016).

The association between lncRNA's role with miRNAs has also been investigated. Angiotensin II (Ang II) is a small polypeptide hormone involved in hypertension. By using RNA-sequencing, 24 novel lncRNAs were identified in Ang II-induced vascular smooth muscle cells (VSMCs). These 24 novel lncRNAs may play a role in mediating cellular responses of VSMCs to Ang II. Furthermore, Lnc-Ang362 functions as a host transcript for two miRNAs (miR-221 and miR-222) implicated in VSMC proliferation. These results provide novel insights into lncRNAs as potential therapeutic targets in Ang II-associated cardiovascular diseases (Leung et al., 2013).

## Summary and prospect

Pulmonary hypertension (PH) is a progressive and severe vasculopathy characterized by vasoconstriction, inflammation, and remodeling of the pulmonary vessels leading to a sustained increase in pulmonary vascular resistance. Epigenetics not only plays an important role in the normal development of human beings, but is also associated with many human diseases. Researches have made great progress of epigenetic knowledge in PH within the recent years. Studies for the role of epigenetics in PH can help to better understand the pathogenesis of PH. In contrast from permanent changes in DNA sequence that causes disease, many epigenetic changes are reversible. This serves as a great potential for therapeutics interventions that may reverse the phenotype of PH.

Based on the understanding of present epigenetics, the majority of the epigenetic drugs are genetically designed to target DNA methyltransferase and histone deacetylase. All other DNA modified proteins may be potential targets for altering the epigenetic status of cells, such as HAT, Histone methylation enzyme and MeCP2 (methyl CpG binding protein 2; Amir et al., 1999). DNA methylation is set and maintained by several kinds of DNA methyltransferase (DNMTs). Nucleoside DNA methyltransferase inhibitors such as 5-azacytidine and Decitabine have shown antineoplasmic activity. However, they also are severely toxic to cells, requiring careful monitoring of the length of time used for and dose administered. Unfortunately, these drugs are not specific toward the type of tissue acted on—causing many unwanted side effects that may outweigh their benefit. Histone modifications play important roles in the regulation of gene expression. Several animal models using HDAC inhibitors have shown to reduce PH (Bogaard et al., 2011; Zhao et al., 2012; Cantoni et al., 2013). These results provide a theoretical basis of HDAC inhibitors for the treatment of PAH. However, there are some limitations and disadvantages in current epigenetic treatments. Broad spectrum HDAC inhibitors will bring about unwanted cardiac side-effects in the treatment for PAH such as TSA (Bogaard et al., 2011). This undesirable effect from nonspecific targets need to be resolved. Selective isoform drugs and their effect on each organ should be studied further. Different types of histone and HDAC isoforms have different distributions in tissues and diseases. With further understanding of their specific roles and expressions, more target- selective drugs could be designed. For PH, some epigenetic abnormalities are not present throughout the genome, thus providing difficulty when changing the epigenetics of a specific gene for treatment. This remains as a challenging problem for therapy. It is meaningful to study the epigenetic mechanisms of key genes or transcription factors, and further explore the corresponding drugs on this basis. Furthermore, whether epigenetic targets act only on the pulmonary vessels or cause an unexpected decrease in systemic blood pressure at the same time, it is an important problem that requires special attention. These HDAC inhibitors are in the preclinical stage, their clinical trials, safety profile, and effects of drugs still need to be well-confirmed before widespread use.

The concept of adult diseases arising from a developmental origin gives many metabolic diseases and pulmonary diseases a reasonable explanation for how they progress in humans. Pregnancy, the neonatal period and childhood are crucial stages for growth and development. Dietary restriction during pregnancy is an important factor affecting the development of the pulmonary arteries. These findings provide significant advancements of epigenetic mechanisms that take effect during pregnancy and infancy for the risk of developing PH. IUGR rats became hypersensitive to hypoxia as a result of higher right ventricular systolic pressure (RVSP) and expression of ET-1. The acetylated histone H3 of the ET-1 promoter of leucocytes were identified in 1-week IUGR rats (Xu et al., 2016), in which the results of ET-1 serve as a biomarker. Early life has great plasticity and is sensitive to the environment. The changes in the neonatal period are reversible and promote the opportunity to intervene with the disease before birth. With effort, we may be able to establish “preemptive medicine” to prevent the development of “the fetal origins of adult diseases.” Future studies need to be conducted to find out the specific components and time points of dietary restriction in childhood. Multiple signaling pathways associated with endothelial cells have been found, but regulatory mechanism of SMC may be more direct. In addition to dietary restriction, other factors such as alcohol, medications, and caffeine are all need to be investigated for their effects on pulmonary vascular development during the perinatal period.

MiRNA and lncRNA are very promising as diagnostic biomarkers, and their analogs or inhibitors may be novel therapeutic targets in the treatment of PH. More epigenetic mechanisms of microRNA and lncRNA have yet to be discovered, and their relationship with DNA methylation and histone modification may provide enough therapeutic potential to consider further research in epigenetics. Currently, the role of microRNA in pulmonary hypertension is based on known genes or pathways, other genes, transcription factors, and pathways that affect pulmonary arterial function still need to be explored. One miRNA may act on hundreds of genes in different organs, and other gene targets need to be avoided in the use of miRNA for treatment. In addition, mechanisms of these miRNAs are based on different PH animal models or patient cells, on different cells (PAEC, PASMC, PH-Fibs). Whether a miRNA has the same effect on different models or cells need further exploration.

The contribution of epigenetic regulation to pulmonary hypertension is an active but complex field of research. The role taken by the epigenetic regulation in the present clinical management of PH reflects the limitations of our current understanding of the disease to some extent. There is ample room for research and diagnostic translation of the pathobiological studies, aimed at procuring the diagnosis and therapeutic power in the epigenetic regulation of pulmonary hypertension.

## Author contributions

All authors listed have made a substantial, direct and intellectual contribution to the work, and approved it for publication.

### Conflict of interest statement

The authors declare that the research was conducted in the absence of any commercial or financial relationships that could be construed as a potential conflict of interest.

## Abbreviations

PH: pulmonary hypertension
EUGR: Extrauterine growth restriction
IUGR: Intrauterine growth retardation
PAH: Pulmonary arterial hypertension
PAEC: pulmonary arterial endothelial cells
PASMC: pulmonary arterial smooth muscle cells
CTEPH: Chronic thromboembolic pulmonary hypertension
DNMTs: DNA methyltransferase
SOD2: Superoxide dismutase-2
HIF1α: hypoxia inducible factor-1α
HAT: histone acetyl transferase
HDAC: histone deacetylases
eNOS: endothelial nitric oxide synthetase
PPHN: persistent pulmonary hypertension of the newborn
DOHaD: Developmental Origins of Health and Disease
ET-1: endothelin-1
hUVEC: human endothelial cells isolated from umbilical veins
IGF-1: Insulin-like growth factor-1
ncRNAs: Noncoding RNAs
BMPR2: bone morphogenetic protein receptor typeII
lncRNAs: Long noncoding RNAs
Ang II: Angiotensin II.
